# HiCoP, a simple and robust method for detecting interactions of regulatory regions

**DOI:** 10.1186/s13072-020-00348-6

**Published:** 2020-07-01

**Authors:** Yan Zhang, Zhaoqiang Li, Shasha Bian, Hao Zhao, Delong Feng, Yanhong Chen, Yuhe Hou, Qifa Liu, Bingtao Hao

**Affiliations:** 1 Department of Hematology, Nanfang Hospital, Southern Medical University, Guangzhou, China; 2 Guangdong Provincial Key Laboratory of Tumor Immunotherapy, Cancer Research Institute, School of Basic Medical Sciences, Southern Medical University, 1023 Shatai Road, Guangzhou, 510515 Guangdong People’s Republic of China; 3 Medical Genetic Institute of Henan Province, Henan Provincial Key Laboratory of Genetic Diseases and Functional Genomics, Henan Provincial People’s Hospital, Zhengzhou University People’s Hospital, Henan University People’s Hospital, Zhengzhou, 450003 Henan China; 4 National Health Commission Key Laboratory of Birth Defects Prevention, Henan Key Laboratory of Population Defects Prevention, Zhengzhou, 450003 Henan China

**Keywords:** Chromatin accessibility, Chromatin structure, Chromatin loop, Enhancer, Promoter

## Abstract

**Background:**

Chromatin physical interactions provide essential information for understanding the regulation of *cis*-elements like enhancers, promoters, and insulators in cell development and differentiation. The Hi-C assay is a technique detecting chromatin structures of the whole genome, but not sensitive to interactions of regulatory elements. Several methods, like HiChIP, DNase-C, and OCEAN-C, have been developed for enriching interactions of regulatory regions, but all of them have some shortcomings. New simple, efficient, and robust methods are still in need for detecting interactions of regulatory regions.

**Results:**

We developed a new, simple, and robust assay called CoP (Column Purified chromatin) for profiling of open chromatin regions by directly purifying fragmentized crosslinked chromatin with a DNA purification column. The accessible chromatin regions, including active enhancers, promoters, and insulators, were significantly enriched in CoP chromatin. The CoP-seq assay can efficiently detect open chromatin regions, especially active promoters, with a high signal-to-noise ratio. We integrated the CoP-seq and Hi-C technique (HiCoP) to detect interactions of accessible chromatin regions, which represent active *cis*-regulatory elements in cells. We observed that the HiCoP captured the peaks in the promoters-associated enhancer regions. HiCoP detected more promoter–enhancer (P–E), promoter–promoter (P–P), and enhancer–enhancer (E–E) interactions within 20 kb–5 Mb than Hi-C. Most of the loops identified by HiCoP are associated with the expressed genes.

**Conclusion:**

CoP assay can efficiently enrich open chromatin regions. When CoP assay was integrated with Hi-C assay, it provides a simple, robust, alternative technique for profiling accessible chromatin regions and chromatin conformation simultaneously.

## Background

The eukaryotic genome is tightly packed in nucleosomes, while some regions are physically accessible. The accessible chromatin regions in the genome provide specific DNA sequences for the binding of transcriptional factors and machinery [1, 2]. The accessible regions are highly dynamic and represent cellular identity [1]. The information on chromatin accessibility is essential for understanding different histone modifications and transcription factor binding in cell development and differentiation. Many methods have been developed for enrichment of open chromatin regions in genome-wide, like ATAC-seq [3], DNase-seq [4, 5], MNase-seq [6, 7], FAIRE-seq [8], etc. The ATAC-seq has emerged as one of the popular methods of genome-wide chromatin accessibility profiling due to its relatively simple procedure and small requirement of cell amount [1, 3].

The spatial chromatin organization, especially the interactions between *cis*-regulatory elements, plays an essential role in the regulation of gene expression by promoting the proximity of promoters and distal enhancers [9, 10]. Chromatin conformation capture (3C) assay is one of the techniques for detecting chromatin interaction by quantifying the proximity of two genome DNA fragments [11, 12]. Combined with deep sequencing techniques, many 3C-based methods like 4C [5, 13, 14], 5C [15], Hi-C [16, 17], Capture-C [18], etc., have been developed for detecting chromatin interactions genome-wide. Hi-C was first described in 2009, where it was used to characterize the chromatin structure of the whole genome [16]. However, Hi-C assay requires very deep sequencing to identify the interactions of regulatory elements fully. To achieve specific chromatin interactions, researchers developed several Hi-C based methods, including Capture Hi-C [18], HiChIP [19], DNase-C [20], Micro-C [21], OCEAN-C [22], etc. The Capture Hi-C is a method of combination of Hi-C and the capture sequencing technique for detecting the interactions of the designed specific genome regions [18]. The HiChIP, a technique similar to ChIA-pet [23], is a combination of chromatin immunoprecipitation (ChIP) with Hi-C to detect the proximity of DNA fragments associated with a specific protein factor [19].

Physical accessibility is a common feature of the regulatory regions, including enhancers, promoters, and insulators [1]. The information on the interactions of the chromatin accessible regions is essential for understanding transcription regulation. Li et al. integrated the FAIRE and the in situ Hi-C assays for mapping global open chromatin interaction, which was named as OCEAN-C, Open Chromatin Enrichment And Network Hi-C [22]. Lai et al. developed a technique (Trac-looping) for analysis chromatin accessibility and interaction at the same time by using bivalent Tn5 [24]. Here, we described a new method of detecting open chromatin by directly purifying sonication-fragmentized crosslinked chromatin with DNA purification column (CoP-seq). We also integrated the CoP-seq and the Hi-C assay to quantify the proximity of the accessible chromatin, which was named as HiCoP. The data showed that HiCoP is a simple and robust method of detecting genome structure and chromatin accessibility.

## Results

**Fig. 1:**
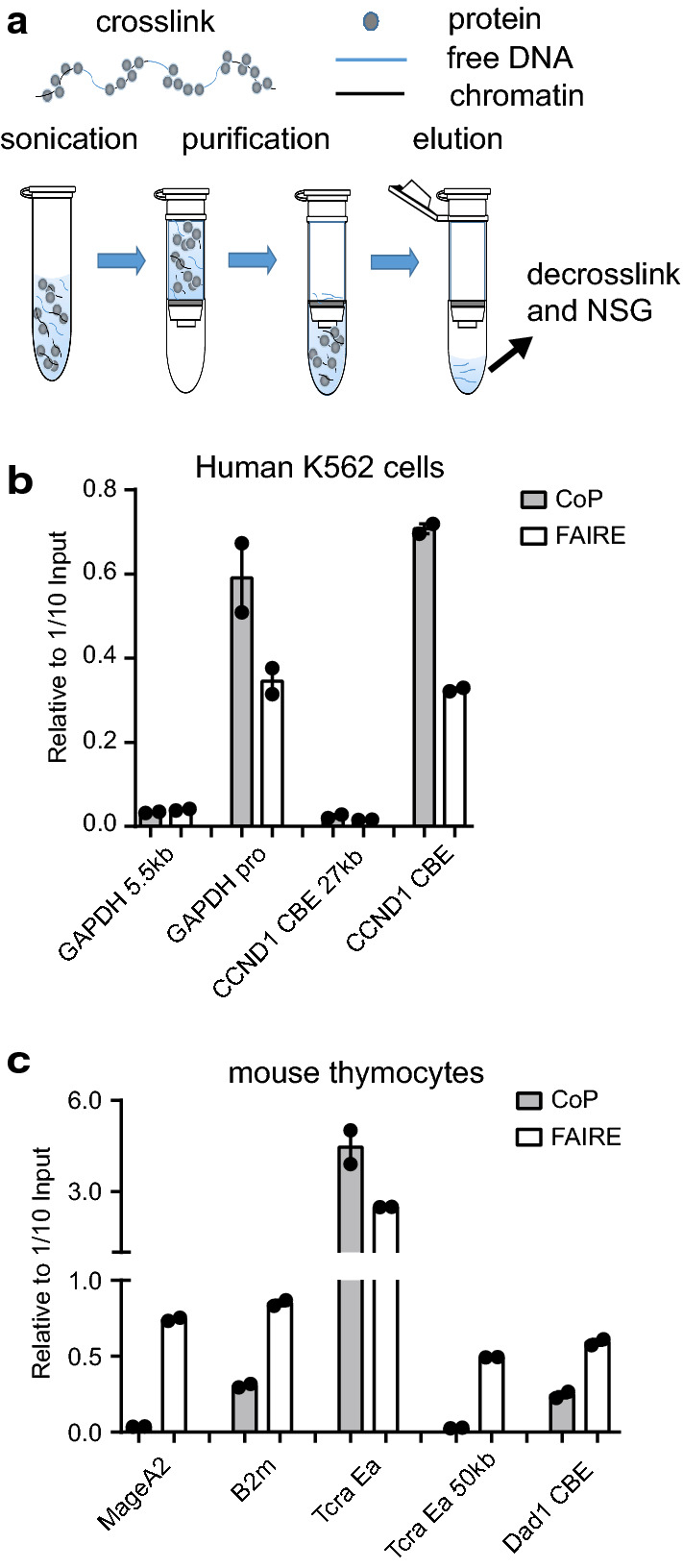
CoP assay enriches regulatory regions. **a** Schematic diagram of CoP assay. **b**, **c** CoP- and FAIRE-qPCR detected the enrichment of regulatory regions in human leukemia cell line K562 cells and mouse thymocytes. GAPDH pro, *GAPDH* gene promoter; GAPDH 5.5 kb, 5.5 kb upstream of *GAPDH* promoter; CCND1 CBE, a CTCF binding site in CCND1 locus; CCND1 CBE 27 kb, 27 kb upstream of the CCND1 CBE; MageA2, *MageA2* gene promoter; B2m, *B2m* gene promoter; Tcra Ea, *Tcra* gene enhancer Eα; Tcra Ea 50 kb, 50 kb downstream of the enhancer Eα; Dad1 CBE, a CTCF binding site in *Dad1* gene. The data are plotted as mean ± SD of two independent experiments

The CoP (Column Purified chromatin) assay takes advantage of the selective binding property of a silica-gel membrane in a DNA purification column, which can effectively and reversibly adsorb naked DNA fragments but not DNA bound with proteins (Fig. 1a). Briefly, after crosslinking with formaldehyde and sonication, cellular chromatin was loaded on a PCR purification column, and the free DNA was purified following the protocol in the PCR Purification kit. To determine whether regulatory regions are specifically enriched in CoP chromatin, we detected an enhancer (T cell receptor gene *Tcra* enhancer Eα in mouse thymocytes), three promoters (active gene *GAPDH* promoter in human K562 cells, active gene *B2m* promoter and silence gene *MageA2* promoter in mouse thymocytes), and two CTCF binding sites (the upstream CBE of the gene *CCND1* in human K562 cells and the CBE of the *Dad1* gene in mouse thymocytes) in CoP chromatin using quantitative PCR (Fig. 1b, c). Active promoters, enhancers, and CTCF binding regions are enriched in CoP chromatin of human leukemia cell line K562 cells and mouse thymocytes, and the enrichments in some regulatory regions are more than 200-fold (Fig. 1b, c). We tested the performance of CoP assay under different formaldehyde concentrations, crosslink time, and sonication strength. All conditions gave high enrichment ratios (Additional file 1: Fig. S1a–c). The CoP assay also has an excellent performance with small amounts of cells (Additional file 1: Fig. S1d) and other types of cell lines and tissues (Additional file 1: Fig. S1e). The results showed that the CoP is a simple and robust technique for detecting open chromatin regions.

Then we did deep sequencing of CoP chromatin purified from K562 cells and thymocytes, with around 22 million unique mapped reads for K562 cells (Additional file 1: Fig. S2a) and 5 million unique reads for thymocytes. After peak calling, we got 22,496 and 21,832 peaks from each replicate of K562 cells and the 18,763 peaks from thymocytes (Additional file 1: Fig. S2a). We compared the CoP peaks with the peaks of ATAC-seq and FAIRE-seq in K562 cells from online data [25]. The CoP assay has a better signal-to-noise ratio (FRiP) than the FAIRE-seq assay (Additional file 1: Fig. S2a). The results showed that the CoP-seq assay is suitable for detection of open chromatin regions.

**Fig. 2:**
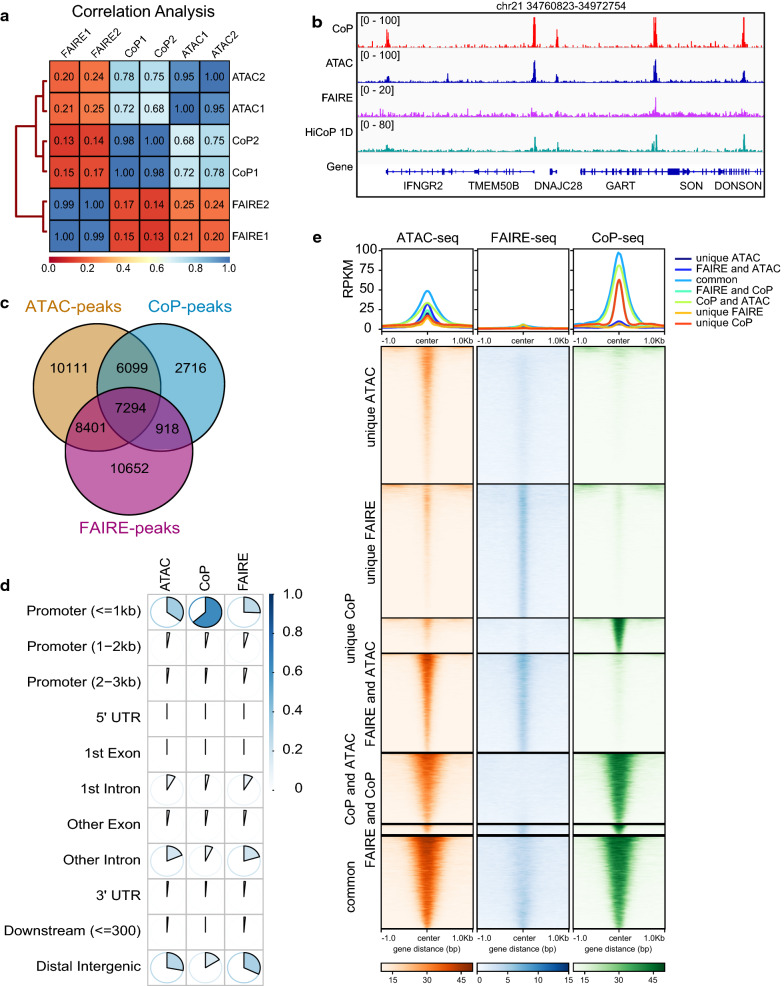
CoP-seq is a robust method for detecting accessible chromatin regions. **a** The correlation analysis of ATAC-seq, CoP-seq, and FAIRE-seq data by using deeptools. **b** The browser view of a 212-kb human genomic region showing the peaks of CoP-seq, ATAC-seq, FAIRE-seq, and HiCoP in K562 cells. **c** Venn diagram of the peaks determined by CoP-seq, ATAC-seq, and FAIRE-seq. It represents the intersected peak numbers from two biological replicates for each method. **d** Comparison of the peak numbers of ATAC-seq, CoP-seq, and FAIRE-seq in promoters, exons, introns, and other regions. **e** Summit-centered heatmaps of the ATAC-seq, FAIRE-seq, and CoP-seq peaks in K562 cells. The unique ATAC peaks were the peaks which weren’t detected by FAIRE-seq and CoP-seq, and so on. The FAIRE and ATAC peaks were the peaks which were not detected by CoP-seq, and so on. The common peaks were detected by the three techniques. The data are from two biological replicates of CoP-seq, ATAC-seq, and FAIRE-seq. ATAC-seq and FAIRE-seq data are from SRA database (ATAC-seq: SRR5809235, SRR5809236; FAIRE-seq: SRR402355, SRR402356)

We found that the CoP-seq data are more similar to ATAC-seq rather than FAIRE-seq, although the procedure of the CoP assay is more similar to FAIRE assay (Fig. 2a, b, Additional file 1: S2b, c). We noticed that 78.6% of the CoP-seq peaks were overlapped with the ATAC-seq peaks while only 42% of ATAC-seq peaks were captured by the CoP-seq (Fig. 2c), which indicates that the CoP-seq may capture a subset of the accessible chromatin fragments. Most of the CoP-seq peaks (63.52%) were located in promoter regions while more ATAC-seq and FAIRE-seq peaks are in intergenic and intron regions (Fig. 2d). To characterize the unique CoP-seq peaks, we compared them with the unique ATAC-seq peaks, the unique FAIRE-seq peaks, the intersected peaks, and the common peaks in the heatmap (Fig. 2e). The common peaks represent a group of peaks with strong signals, while the signals of the unique CoP-seq peaks were weaker. The functional annotation of the peaks showed that most of the common peaks (73%) were associated with active promoters (Additional file 1: Fig. S2d). Most of the unique CoP peaks were located at active promoters (34%), weak promoters (27%), and weak enhancers (22%). The CoP-seq data showed that CoP chromatin mainly represents active promoters, suggesting that the CoP-seq assay can be combined with the Hi-C technique for detecting promoter–enhancer interactions.

**Fig. 3:**
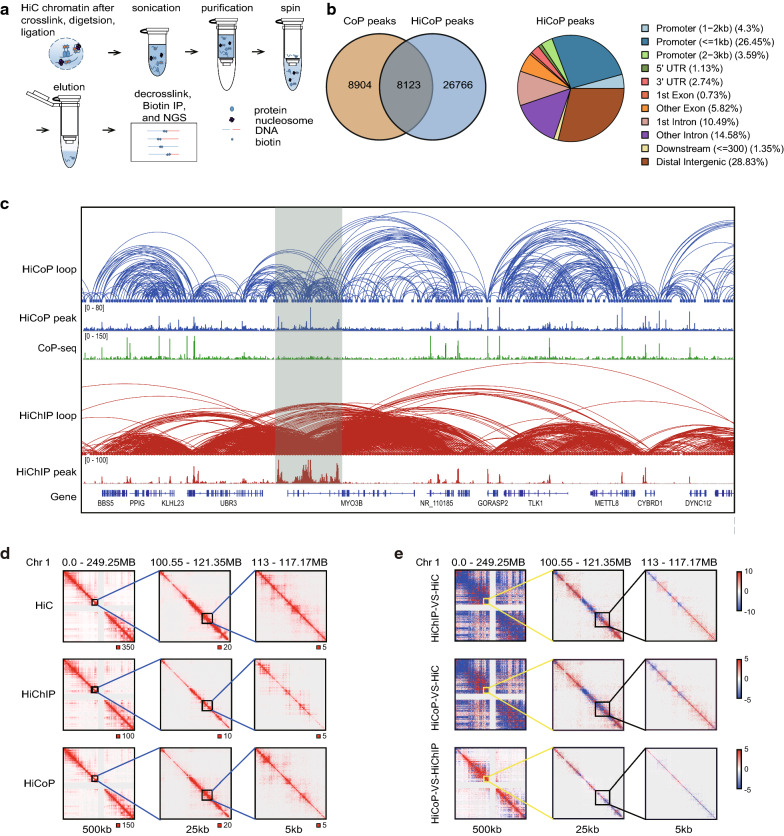
HiCoP efficiently detects open chromatin interactions. **a** Schematic diagram of HiCoP assay. **b** Venn diagram of the peaks of CoP-seq and HiCoP (left) and peak annotation of HiCoP assay (right) in K562 cells. **c** HiCoP 3D, HiCoP 1D, CoP, HiChIP 3D, and HiChIP 1D signal enrichment at MYO3B locus in IGV. PETs are called from two replicates by using HiC-Pro. Loops are enriched in MYO3B enhancer (highlighted in gray). **d** Heatmaps and **e** subtraction heatmap of HiCoP, HiChIP (H3K27ac), and HiC on chromosome 1. The numbers below the heatmaps are resolution. The interaction matrix was displayed in juicerbox. The data were normalized by using the Knight–Ruiz balancing. Red represents positive value, blue negative value. HiC and HiChIP(H3K27ac) data are downloaded from SRA database (HiC: SRR1658693, SRR1658694; HiChIP: SRR5831492, SRR5831493)

We generated HiCoP libraries of K562 cells (two biological duplicates) by adding purification of CoP chromatin in the Hi-C procedure and got a total of 0.6 billion clean reads (Fig. 3a). We obtained around 182 million unique PETs after mapping, and most of the PETs (84%) are *cis*-interaction (Additional file 1: Fig. S3a). To address enrichment specificity, we identified the peaks from the HiCoP data. We called a total of 34,889 common peaks using MACS2 peak calling followed by median absolute deviation filtering. 47.7% of CoP peaks overlapped with HiCoP peaks, and 23.3% HiCoP overlapped with CoP (Fig. 3b). 34.2% of the HiCoP peaks are located at promoter regions, which is less than 63.52% of the CoP-seq peaks (Figs. 2d and 3b). More HiCoP peaks are located in intergenic regions (28.83%) compared with the CoP-seq (Fig. 3b). We observed that the HiCoP captured the peaks in the promoters-associated enhancer regions, which were not detected by CoP-seq (Fig. 3c). The heatmap showed that the unique HiCoP peaks were wide and weak peaks, and most of the peaks were located at enhancer region (Additional file 1: Figs S3b and S3c). The change in peak distribution of HiCoP may be explained by co-capturing regions associated with CoP DNA fragments like enhancers.

Next, we compared the interaction maps identified by HiCoP with HiChIP of H3K27 acetylation, a histone marker of accessible chromatin region, and Hi-C in the same cell lines [17, 26]. We generated 500-, 25- and 5-kb-resolution read-normalized interaction maps in HiCoP, HiChIP of H3K27ac, and Hi-C (Fig. 3d). We observed some regions with reduced interactions in the 5-kb-resolution HiCoP heatmap compared with that in the Hi-C heatmap, which was also seen in the HiChIP heatmap (Fig. 3d). To better understand the similarity and difference between HiCoP and HiChIP, we did signal subtractions of HiChIP-Hi-C, HiCoP-Hi-C, and HiCoP-HiChIP (Fig. 3e). We noticed that HiCoP detected more interactions in the active regions than Hi-C, which is similar to HiChIP (Fig. 3e). In the 500-kb-resolution heatmap, there are fewer long-distance interactions identified by HiCoP and HiChIP than Hi-C. HiCoP heatmap displays less long-distance interactions than HiChIP in 500-kb resolution, but the difference was not obvious in the 25- and 5-kb-resolution maps (Fig. 3e).

**Fig. 4:**
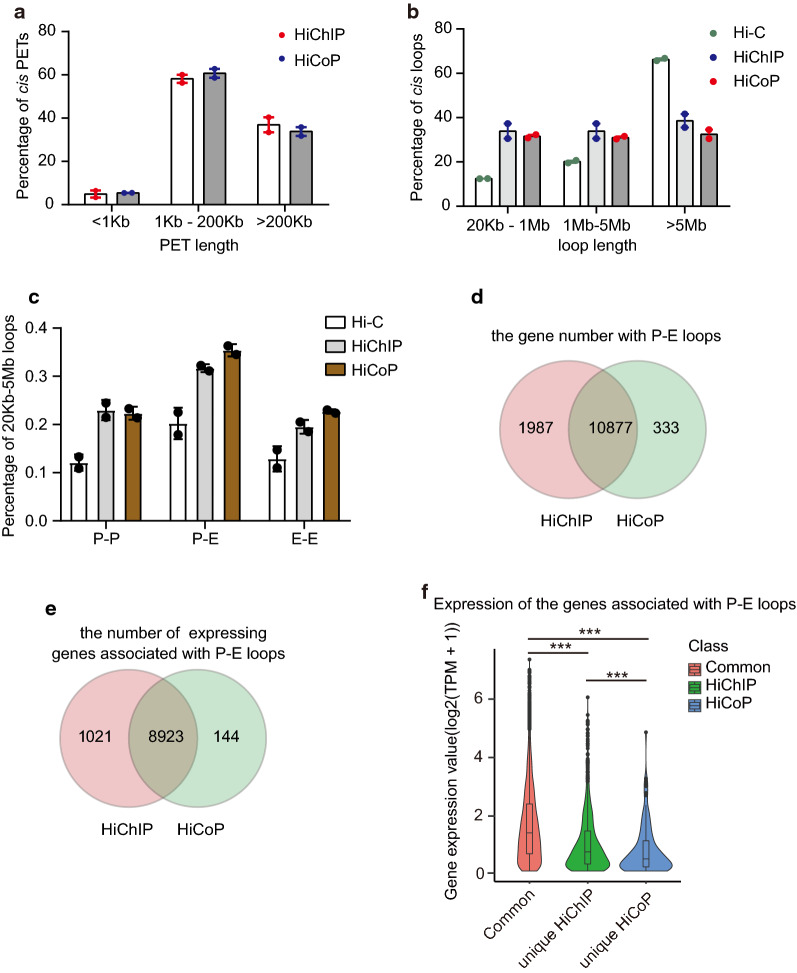
HiCoP has an advantage in detecting promoter–enhancer interactions. **a** Distribution of the *cis-*PETs of HiChIP and HiCoP. PETs are called from two replicates by using HiC-Pro. **b** Distribution of the *cis* loops of HiC, HiChIP, and HiCoP. Loops were called from two replicates by using Fit-Hic. **c** Distribution of the functionally annotated loops. P–P, loop between promoter and promoter; P–E, promoter and enhancer; E–E, enhancer and enhancer. **d** Venn diagrams of the genes associated with P–E loops and **e** the K562-expressing genes associated with P–E loops. K562 RNA-seq data are from ENCODE database, and expression cut level is TPM > = 0.5. **f** The expression levels of the K562-expressing genes associated with P–E loops. *P* values were calculated by Wilcoxon test. *** < 0.001. HiC and HiChIP(H3K27ac) data were downloaded from SRA database (HiC: SRR1658693, SRR1658694; HiChIP: SRR5831492, SRR5831493)

We found that 95% of the *cis*-PETs are > 1 kb and around 60% are within the range of 1–200 kb, and more than 30% are > 200 kb, which is similar to the distribution of the HiChIP *cis*-PETs (Fig. 4a). Most of the promoter–enhancer interactions are in the range of 1–200 kb, and the > 200 kb PET length reveals the higher-order chromatin organization. The *cis*-PETs length distribution of the HiCoP data indicates its ability in measuring the enhancer–promoter interactions. Then we called loops from HiCoP, HiChIP, and Hi-C data by using FitHiC and pgltools. We got 18,601,021 loops from HiCoP, 21,919,151 from HiChIP, 78,326,558 from Hi-C. Around 33% HiCoP loops are within the range between 20 kb and 1 Mb, 33% within 1–5 Mb, 33% > 5 Mb. HiChIP loops are pretty similar to HiCoP, while more Hi-C loops are > 5 Mb (Fig. 4b).

To explore the functional significance of the loops identified by HiCoP, HiChIP, and Hi-C, we annotated the loops with enhancer and promoter information of the K562 cells from ChromHMM data. There are two types of strong enhancers in ChromHMM: strong enhancer four and strong enhancer five. Both types of strong enhancers have H3K4me3 and H3K27ac, but the strong enhancer four has H3K4me1 and higher H3K9ac, and the strong enhancer five has no H3K4me1 and lower H3K9ac. We merged the two types of enhancers for enhancer–promoter interaction analysis. We found that HiCoP detected more promoter–enhancer (P–E), promoter–promoter (P–P), and enhancer–enhancer (E–E) interactions within 20 kb–5 Mb than Hi-C, which is similar to HiChIP (Fig. 4c). 97% of the genes with the P–E loops identified by HiCoP are overlapped with that by HiChIP, and around 85% of the HiChIP P–E loop-associating genes were detected by HiCoP (Fig. 4d). The results suggested that the HiCoP loops represented a major subset of the HiChIP loops. To explore the relationship between the P–E loops and gene expression, we analyzed the expression of the genes with P–E loops in K562 cells (Fig. 4e). More than 80% of the genes associated with the common P–E loops are expressed in K562 cells, which are slightly higher than the ratio (77%) of the P–E loops identified by H3K27ac HiChIP (Fig. 4e). We also analyzed the expression level of the genes associated with loops (Fig. 4f). The common loop-associating genes represented a group of highly expressed genes. The result showed that the P–E loops identified by the HiCoP were associated with the highly expressed genes in K562 cells.

## Discussion

Here we provided a simple and robust method, CoP assay, for the detection of accessible chromatin regions. The principle of the Cop assay is similar to the FAIRE assay [27]: both methods use sonicated formaldehyde-crosslinked chromatin for nucleosome-free DNA. The difference is that the CoP assay purifies accessible chromatin by using a DNA purification column instead of phenol–chloroform in the FAIRE assay, which makes the procedure of the CoP assay simpler than the FAIRE. Moreover, the CoP-seq has a lower background than the FAIRE-seq, which may be due to the sensitivity of DNA purification columns to protein-bound DNA. The CoP-seq has the common disadvantage of the accessible chromatin detection method, which is that it is sensitive to dead cells. However, unlike the ATAC-seq assay [28], the CoP-seq does not capture a lot of mitochondrial DNA.

Most of the CoP-seq peaks are located at active promoter regions, which represent a subset of the accessible chromatin regions. Interestingly, the HiCoP detected more promoter-associated enhancers, which makes the HiCoP able to measure active promoters and its associated enhancers. The histone modification H3K27 acetylation is an essential marker of active promoters and enhancers. So the H3K27ac HiChIP has an advantage in detecting the promoter–enhancer interactions. The HiCoP has a comparable ability in detecting the promoter–enhancer interactions while the procedure is much simpler than the HiChIP. OCEAN-C is a FAIRE-based technique of detecting open chromatin interactions [22]. We did not compare HiCoP and OCEAN-C due to the lack of OCEAN-C data from K562 cells. We noticed that the number of the OCEAN-C 1D peaks was much less than the FAIRE-seq peak number (around 17%) [22], while the HiCoP 1D peak number is higher than the CoP-seq peak number. It suggested that HiCoP is more sensitive to open chromatin regions. In addition to the techniques developed for measuring interactions of regulatory regions like HiChIP [19], OCEAN-C [22], and Trac-looping [24], here we provided a simple, robust, alternative technique HiCoP for profiling accessible chromatin regions and chromatin conformation simultaneously.

## Conclusion

CoP assay can efficiently enrich open chromatin regions. When CoP assay was integrated with Hi-C assay, it provides a simple, robust, alternative technique for profiling accessible chromatin regions and chromatin conformation simultaneously.

## Methods

### Cell culture

Jurkat cells (ATCC CRL-2901) were grown in RPMI-1640 medium containing 10% fetal bovine serum at 37 °C and 5% CO_2_. K562 cells (ATCC CCL 423) were grown in IMDM medium containing 10% fetal bovine serum at 37 °C and 5% CO_2_, and HL-60 cells (ATCC CCL-240) were grown in IMDM medium containing 20% fetal bovine serum at 37 °C and 5% CO_2_.

### Mouse

Wild-type C57BL/6 mice were purchased from Guangdong Medical Animal Experimental Center and housed in a specific pathogen-free facility managed by the Southern Medical University Division of Laboratory Animal Center. Thymus, liver, and kidney from 4–12 weeks C57BL/6 WT mice were ground in MACS buffer (1× PBS, 0.5% BSA, 2 mM EDTA) and filtered through a 40-μm nylon mesh. Cells were centrifuged at 1200 rpm for 5 min at 4 °C. Red cells were lysed in 2–4 ml of AcK buffer (0.15 M NH4Cl, 10 mM KHCO3, 0.1 mM EDTA, adjust PH to 7.4) for 5 min at room temperature. Then 10 ml MACS buffer was added to quench AcK, centrifuged at 1200 rpm for 5 min at 4 °C. Cells were resuspended at 1 ml of cold 1× PBS, counted cell, taken out 10^7^ cells. For crosslinking cells, formaldehyde was added at a final concentration of 1% at RT for 10 min, and then quenched with glycine (0.125 M) for 5 min. The crosslinked cells were washed once with 1 × PBS, followed with CoP assay or flash-frozen by liquid nitrogen, and stored at − 80 °C for further use.

### CoP procedure

The cells were cultured to 80–90% confluence and then collected, 1 × 10^7^ cells were centrifuged at 1,500 rpm for 5 min at room temperature (22–25 °C). The pellet was resuspended in 5 ml of 1× PBS with 20% FBS (fetal bovine serum). 5 ml of 4% formaldehyde was added in 1× PBS, and mixed thoroughly by inverting 4 ~ 6 times, then leave on bench for 10 min. 500 μl of 2.5 M Glycine was added to quench crosslinking reaction for 5 min on bench. Cells were centrifuged at 1,800 rpm for 5 min at 4 °C and supernatant was removed. Cells were washed once with 1 ml of cold 1× PBS, and then harvested by centrifugation at 1,800 rpm for 5 min at 4 °C. The pellet was resuspended in 1 ml cold lysis buffer (1% Triton X-100, 0.5% NP-40, 150 mM NaCl, 50 mM Tris–Cl at pH 7.5, 5 mM EDTA) with 1× PIC (Proteases Inhibitor Cocktail, Roche, 4693132001) and incubated for 10 min on ice. Nuclei were centrifuged at 2000 rpm for 5 min at 4 °C. Pellet was resuspended in 200 μl nuclei lysis buffer (50 mM Tris–Cl at pH 8.0, 10 mM EDTA, 1% SDS), incubated for 10 min on ice. Cells were centrifuged at 2000 rpm for 5 min at 4 °C. Pellet was resuspended in 400 μl of cold 1× TE and transferred to a 0.6-ml fresh tube. Then samples were sonicated for 30 min at 15 s on/25 s off with a Q800R2 sonicator at 60% amplitude. 40 μl of sample chromatin was spared as input, 360 μl of chromatin was purified by PCR purification kit (Dong Sheng, China, N1093), and eluted to 40 μl EB as CoP chromatin. 0.5 μl of 100 mg/ml RNase A was added to input and CoP sample and incubated at 37 °C for 30 min. Then 5 μl of 10 mg/ml proteinase K (Invitrogen, 25530015) was added and incubated at 55 °C for 30 min, then 65 °C overnight. DNA was purified with a PCR purification kit and eluted in 50 μl EB. For a small amount of cells CoP, 10^6^, 10^5^, 10^4^, 10^3^ of cells were taken out as the proportion after sonication.

### FAIRE

FAIRE was performed as the procedure published before [8]. Briefly, crosslinking and sonication was done as CoP. After sonication, half of the chromatin was taken out for CoP, half for FAIRE. DNA was isolated by adding an equal volume of phenol (Solarbio, T0250), vortexing, and spinning at 15,000 rpm for 5 min at RT. The aqueous phase was isolated and stored in a separate tube. An additional equal volume of TE was added to the organic phase, vortexed, and spun again at 15,000 rpm for 5 min at RT. An equal volume of phenol–chloroform (Solarbio, T1012) was added, then vortexed and spun at 15,000 rpm for 5 min at RT. The aqueous phase was transferred to a fresh tube. An equal volume of chloroform (amresco, 0757-500ML) was added to the aqueous phase, then vortexed and spun again. For decrosslinking, 1 μl of 100 mg/ml RNase A was added to the sample and incubated at 37 °C for 30 min. Then 5 μl of 10 mg/ml proteinase K was added to each tube and incubated at 55 °C for 30 min, followed with incubation at 65 °C overnight. DNA was purified by PCR purification kit and eluted in 50 μlEB.

### Quantitative PCR

QPCR was performed using HieffTM qPCR SYBR Green Master Mix (YEASEN, 11203ES08) on an ABI step one plus real-time PCR instrument. Relative enrichment of each amplicon in CoP or FAIRE DNA was calculated by dividing the active region by the inactive region. DNA from untreated cells served as the control for the calculations.

### NGS Library preparation

The sequencing libraries were prepared by either Tn5 tagmentation or linker ligation. Tagmentation was done with a TransNGS Tn5 DNA Library Prep Kit for Illumina (Trans, KP101-01) following the manufacturer’s recommendations. The reaction was incubated 5 min at 55 °C followed by adding 30 μl of × Tn5 Digestion Mix and incubated 5 min at 55 °C, then purified with MagicPure Size Selection DNA Beads (Trans, EC401) and elution in 21 μL of water. After that, the library (20 μL) was amplified by using TransNGS Library Amplification SuperMix. Alternatively, end repair and dA tailing were done with VAHTSTM Universal End preparation Module for Illumina (Vazyme, N203-01/02). DNA was purified with VAHTS DNA Clean beads (Vazyme, N411-01-AA). Added adapter by Quick ligase (NEB, M2200L), then the library was amplified with a PCR amplification kit (Vazyme, P515-02). After 5 cycles of amplification, qPCR (YEASEN, 11203ES08) was done to determine the additional cycle number. After library amplification, purification, and size selection, library concentration was detected by Qubit (Invitrogen, Q32854). The libraries were sequenced by HiseqXten-PE150 Illumina sequencing platform in Novogene Corporation Inc.

### Analysis of CoP-seq, ATAC-seq, and FAIRE-seq data

High-confidence reads of CoP-seq data obtained by using fastp with default parameters, were mapped to human genome version hg19 or mouse genome mm10 by using Bowtie2 with parameters (–sensitive, –X 2000), and PCR duplicated fragments were filtered by Picard [29]. Peaks were identified by MACS2 with parameters (–nomodel, –extsize 147, –broad and –broad-cutoff 0.1). FRiP (fragments ratio in peaks) value was calculated by using bedtools and awk. We used deepTools to generate bigWig file with RPKM normalization. Peaks were annotated by using R package ChIPSeeker [30]. The same procedures were done for ATAC-seq and FAIRE-seq data.

### HiCoP procedure

HiCoP was performed with a modification of the HiC procedure [17]. Briefly, 10^7^ crosslinked cells were digested with MboI (NEB, R0147). Then filling-in with biotin-labeled dCTP and re-ligation by the T4 ligase was performed. After washing, cells were resuspended in 400 μl of cold 1 × TE and sonicated for 20 min at 15 s on/25 s off using a Q800R2 sonicator at 60% amplitude. 40 μl of chromatin was spared as input, and the remaining was purified by using a PCR purification kit (Dong Sheng, N1093), and eluted to 40 μl EB. For decrosslinking HiCoP chromatin, 1 μl of 100 mg/ml RNase A was added to the input and CoP chromatin and did incubation at 37 °C for 30 min. 5 μl of 10 mg/ml proteinase K was added and incubated at 55 °C for 30 min, then 65 °C overnight. DNA was purified by PCR purification kit and eluted in 50 μl EB. The concentration of DNA was measured by using Qubit. 5 μl of 10 mg/ml Dynabeads MyOne Streptavidin C1 beads (Invitrogen, 65001) was washed once with 200 μl of 1× Tween washing buffer (1× TWB: 5 mM Tris–HCl pH 7.5, 0.5 mM EDTA, 1 M NaCl, 0.05% Tween 20). The beads were resuspended in 50 μl of 2× binding buffer (10 mM Tris–HCl pH 7.5, 1 mM EDTA, 2 M NaCl). The beads were added to HiCoP DNA and incubated at RT for 15 min with rotation. The beads were washed by adding 200 μl of 1 × TWB. The tubes were heated on a Thermomixer at 55 °C for 2 min with mixing. The beads were reclaimed using a magnet, and supernatant was discarded. Repeat washing. End-repair and dA tailing following the in situ Hi-C protocol, adapters were added to DNA fragments. PCR amplification was performed with 13 to 19 cycles using Illumina primers. Finally, DNA size selection was performed with 0.55–0.75 × volume of VAHTS DNA Clean beads (Vazyme, N411-01-AA). The library was quantified with Qubit and sequenced using Novaseq-PE150 Illumina sequencing platform at Berry Genomics Corporation Inc.

### Data analysis of HiCoP, HiChIP, and HiC

Clean reads were mapped to human genome (hg19) by HiC-Pro with default parameters [31]. Interaction heatmaps of HiC, HiChIP, and HiCoP were generated by Juicebox with hic file transferred from HiC-Pro matrix by HiC-Pro. PETs were derived from valid interaction of the HiC-Pro result. bigWig files were generated from HiC-Pro result by using deepTools. HiCOP and HiChIP interaction loops were detected by using FitHiC at 10k resolution with parameter (-p 2, -L 20000, -× All, -noOfBins 100), significant interaction loops were defined by q < 0.05. Loops were annotated with UCSC functional DNA elements and encode expressed genes by pgltools.

## 1D enrichment peak analysis

Paired-end bam files were transferred to single-end bam file, and then broadpeaks were generated by MACS2 with default parameters, and high reliable peaks were identified as outliers calculated by median absolute deviation (MAD).

## Supplementary information

**Additional file 1: Additional figures.**

## Data Availability

CoP-seq and HiCoP data are available in GSE database as GSE144412. ATAC-seq (SRR5809235, SRR5809236), FAIRE-seq (SRR402355, SRR402356), HiChIP (SRR5831492, SRR5831493) and HiC (SRR1658693, SRR1658694) of K562 SRA data were download from SRA database. ChromHMM data: Human genome (hg19) chromatin-state signatures file produced by ChromHMM was downloaded from UCSC (http://hgdownload.soe.ucsc.edu/goldenPath/hg19/encodeDCC/wgEncodeBroadHmm/wgEncodeBroadHmmK562HMM.bed.gz). K562 mRNA expression matrix was downloaded from encode database (ENCFF558HFV). The most representative promoter of human (16,455 genes) gene from EPDnew database.
