# Supplementary material for: HiCoP, a simple and robust method for detecting interactions of regulatory regions
Source: Epigenetics Chromatin. 2020 Jul 1;13:27. doi: 10.1186/s13072-020-00348-6 (PMC7329539; doi:10.1186/s13072-020-00348-6)
Supplement: Supplementary file 1 — Additional file 1: Additional figures. [file 13072_2020_348_MOESM1_ESM.docx]

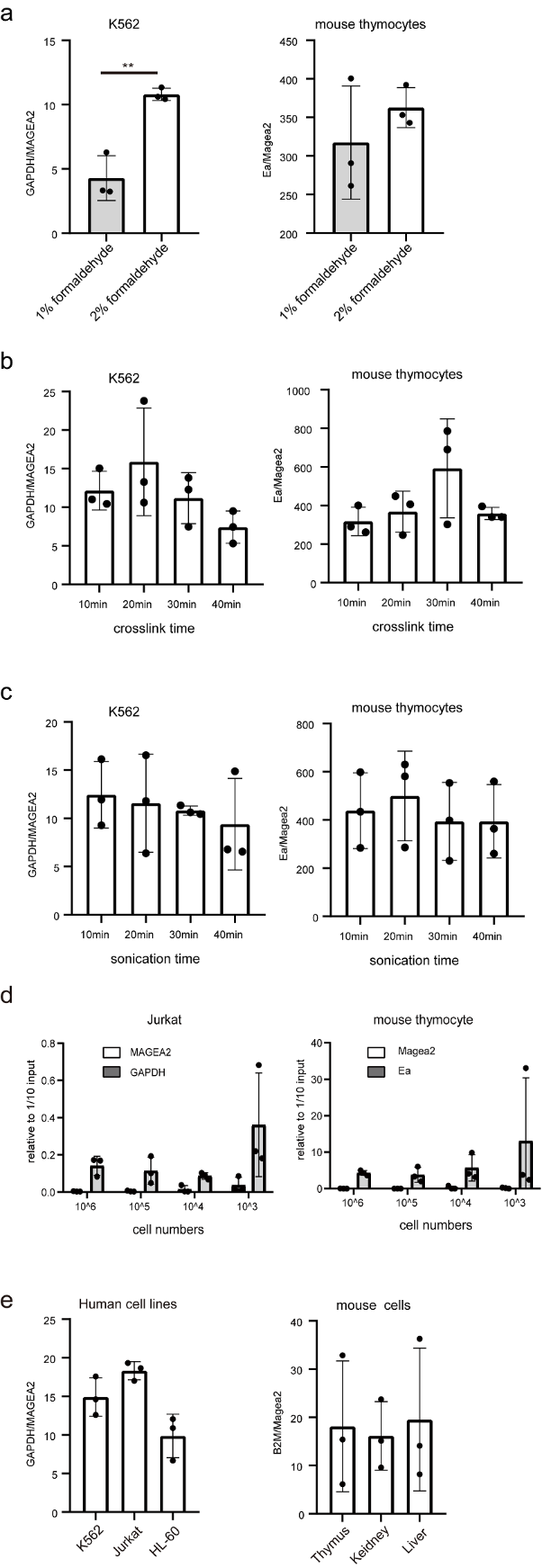


**Fig. S1 CoP assay can robustly detect accessible chromatin.** a-c) Enrichment of active regions and inactive regions in two formaldehyde concentrations (a), four crosslink times at 1% formaldehyde concentration (b), four sonication times (c). d) CoP chromatin enrichment in low-cell-numbers. e) CoP enrichment in three human cell lines and three mouse tissues. GAPDH, GAPDH gene promoter; Magea2, Magea2 gene promoter; Ea, Tcra gene enhancer Eα; B2M, B2m gene promoter. The common CoP condition: crosslink with 2% formaldehyde for 10 minutes and sonication for 30 minutes. The data are plotted as mean ± SD of three independent experiments.


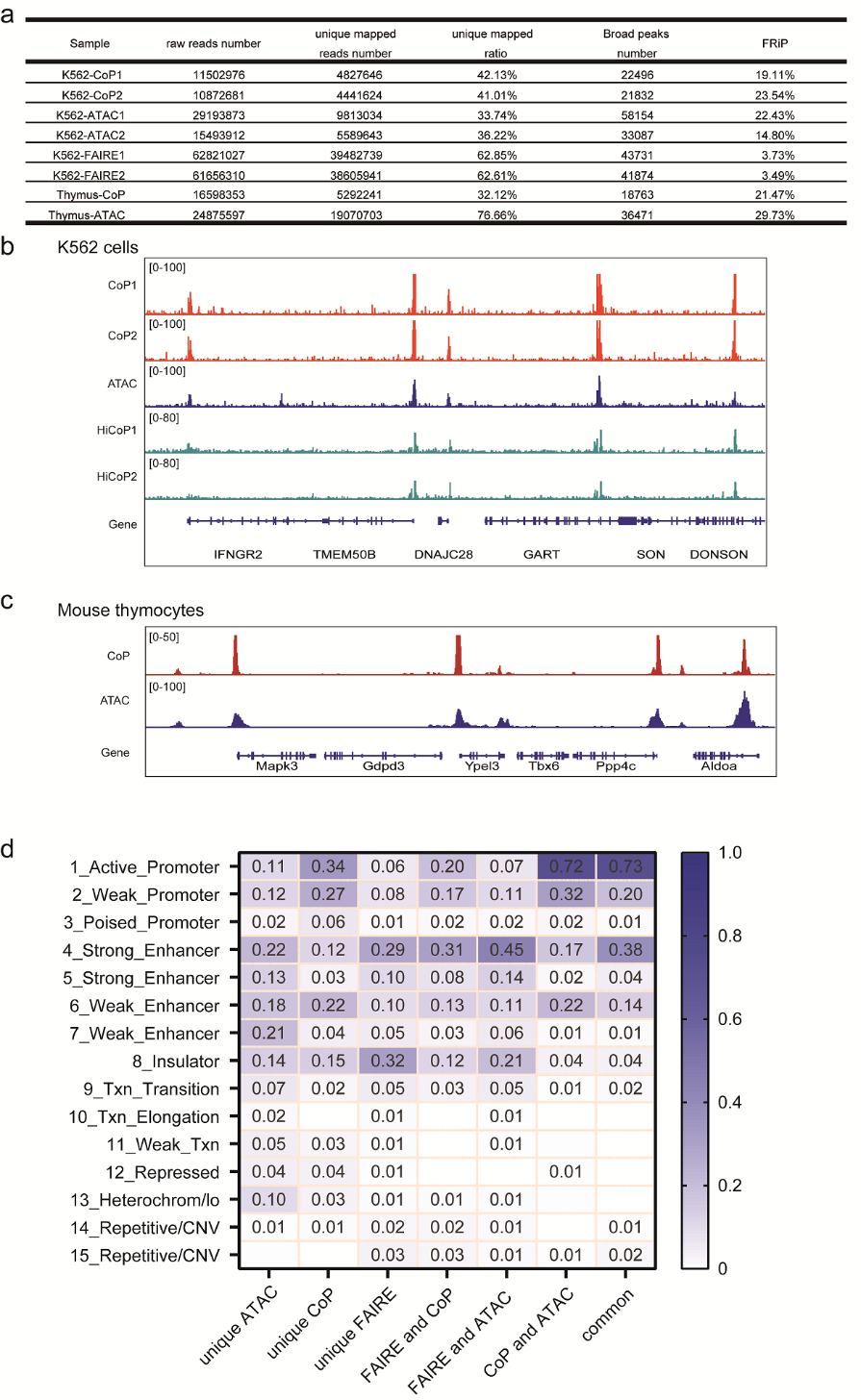


**Fig. S2 CoP-seq detects accessible chromatin regions in genome-wide.** a) Deep sequencing information of CoP-seq, ATAC-seq, and FAIRE-seq. FRiP, Fragment Ratio in Peaks. b) Signal tracks of CoP-seq, ATAC-seq, and HiCoP of K562 cell. c) Signal tracks of CoP-seq and ATAC-seq of mouse thymocytes. d) The functional annotation of the ATAC-seq, FAIRE-seq, and CoP-seq peaks in K562 cells. Each cell contains the ratio of the annotated peaks in each type of peaks (normalized to 1). Each assay contains two biological replicates.


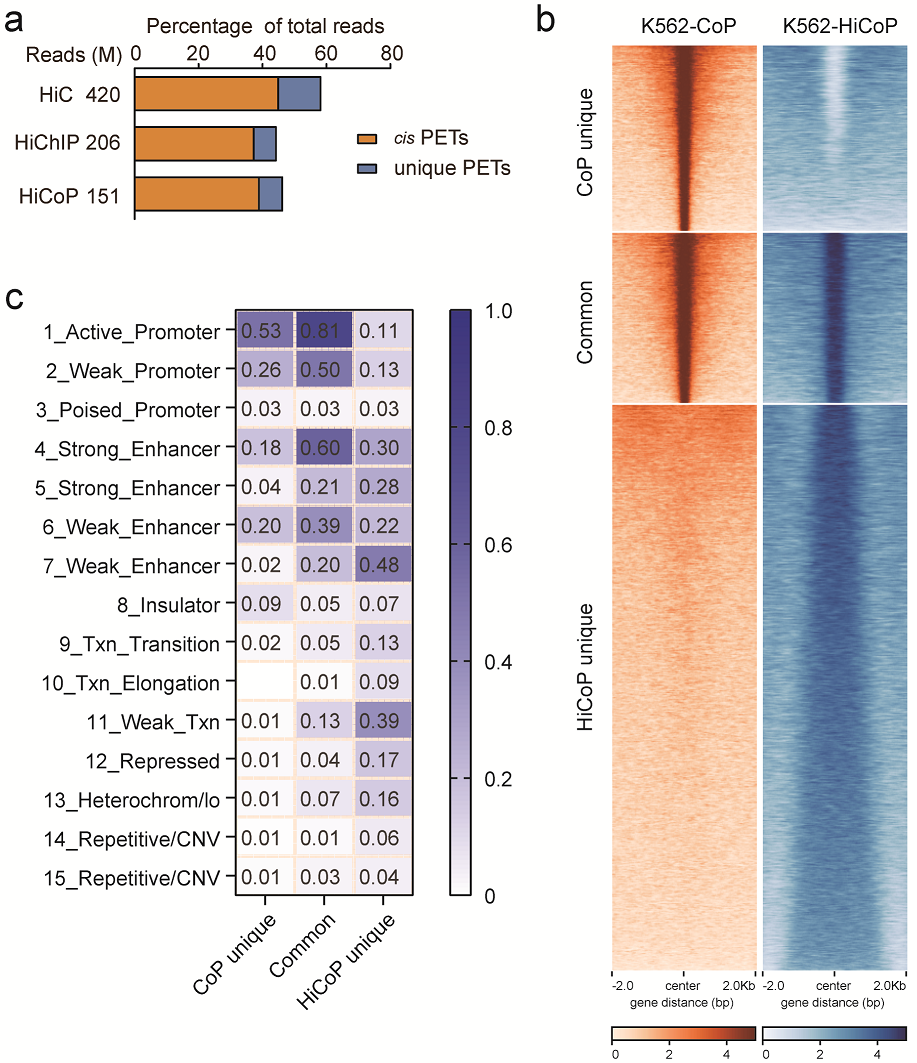


**Fig. S3 HiCoP efficiently detects interactions of regulatory regions.** a) PET information of one example of HiChIP and HiCoP assay of K562 cells. b) Summit-centered heatmaps of the CoP-seq and HiCoP 1D peaks in K562 cells. c) The functional annotation of the CoP-seq and HiCoP 1D peaks in K562 cells.
